# Seismic hazard prediction of the Hunhe Fault in the Shen-Fu New District

**DOI:** 10.1038/s41598-024-64946-0

**Published:** 2024-06-25

**Authors:** Zijun Wang, Boming Zhao, Bo Wan

**Affiliations:** 1 Key Laboratory of Urban Underground Engineering of Ministry of Education, Beijing Jiaotong University, Beijing, 100044 China; 2 School of Civil Engineering, Beijing Jiaotong University, Beijing, 100044 China; 3 Earthquake Administration of Liaoning Province, Shenyang, 110034 China

**Keywords:** Active fault, Strong ground motion estimation, Seismic hazard, Seismic code, Shen-Fu New District, Solid Earth sciences, Seismology

## Abstract

Earthquake prevention and disaster mitigation are crucial aspects of social welfare that significantly impact national public security. This paper presents a seismic risk assessment and hazard prediction of the Hunhe Fault in the Shengyang-Fushun (Shen-Fu) New District. The target area is at risk of seismic damage due to two major branch ruptures, namely, F9 and F1; these ruptures have the potential to generate maximum earthquakes with a magnitude of 6.0 in the next 50 to 100 years. A three-dimensional underground velocity structure and asperity source model were established for the target faults. Subsequently, a hybrid technique combining deterministic and empirical approaches was employed to simulate the broadband strong ground motion of the target region in anticipation of the occurrence of expected scenario earthquakes. The distributions of peak ground acceleration (PGA), peak ground velocity (PGV) and peak ground displacement (PGD) for the area are provided, and the results indicate that densely populated urban areas could experience PGA values close to 280 cm/s^2^ along the fault traces. This study provides a reliable basis for engineering construction and urban planning in the Shen-Fu New District.

## Introduction

Earthquake prevention and disaster reduction represent a fundamental and crucial aspect of social welfare, with a significant impact on national public security. With the expansion of urban areas and the sustained development of the economy, the losses caused by earthquakes are also increasing^1–3^. Consequently, the effective implementation of measures to prevent and mitigate earthquake disasters and create a safe and hospitable living environment has become a major issue of concern. A strong correlation exists between active faults and seismic activity. Earthquakes and their resulting hazards cause the greatest disaster and loss of life in regions located alongside and near faults^4,5^. Studies and investigations into active tectonics are vital for identifying the exact location, scale, and movement characteristics of target ruptures, along with their level of activity, segmentation, and potential seismic hazard. This scientific foundation supports mid- to long-term earthquake prediction and prevention, disaster mitigation strategies, and effective measures to prevent future earthquakes triggered by active faults^6^.

The Tanlu Fault Zone is a giant tectonic rupture zone situated in the eastern part of China and extends northwards through the Bohai Sea and the Lower Liaohe Plain to the southwest of Shenyang. This has resulted in the formation of two primary branching ruptures: the Yilan-Yitong Fault and the Mishan-Dunhua Fault^7,8^. These rupture zones constitute the most significant seismic tectonics that impact the study area. The Liaoning segment of the Mishan-Dunhua Fault, also known as the Hunhe Fault, originates from southern Shenyang and traverses through Fushun and Qingyuan before being offset by the Chifeng-Kaiyuan Fault near the border of Liaoning and Jilin Provinces. The fault has an overall length of approximately 200 km and strikes towards the northeast-east. The Hunhe Fault comprises two to three nearly parallel compression and torsional fractures, along with their subbranches; these are separated by distances of 1–2 km to 10–20 km. The main fracture zone is considerable in size, with a width of up to 50 to 60 m. The Hunhe Fault zone is situated beneath urban areas and has a significant impact on construction, geology and earthquake safety.

In this paper, the risk assessment and seismic hazard prediction of the Hunhe Fault in the Shen-Fu New District are analysed. The major fault ruptures that present seismic risk to the area have been identified, and the potential maximum magnitude within the next 50 to 100 years has been estimated. This estimation was made by accounting for the investigated tectonic stress conditions, deformation characteristics, late Quaternary activity levels, and deep geological structures and geophysical features. Subsequently, a three-dimensional velocity structure and asperity source model for the target faults was established. Finally, a hybrid technique combining deterministic and empirical approaches was employed to simulate strong ground motion in the target area caused by scenario earthquakes. Accordingly, the distributions of peak ground acceleration (PGA), peak ground velocity (PGV) and peak ground displacement (PGD) for the area were obtained. The results provide a scientific basis for the assessment of seismic safety, planning of urban development, management of land use, selection of project sites, and design of buildings in areas prone to earthquakes.

## Seismic risk analysis of the target faults

The Shen-Fu New District is a national reform and innovation demonstration zone strategically located between the cities of Shenyang and Fushun in Liaoning Province, Northeast China. The district is intended to serve as a new engine for the revitalisation and development of Liaoning Province.

**Figure 1:**
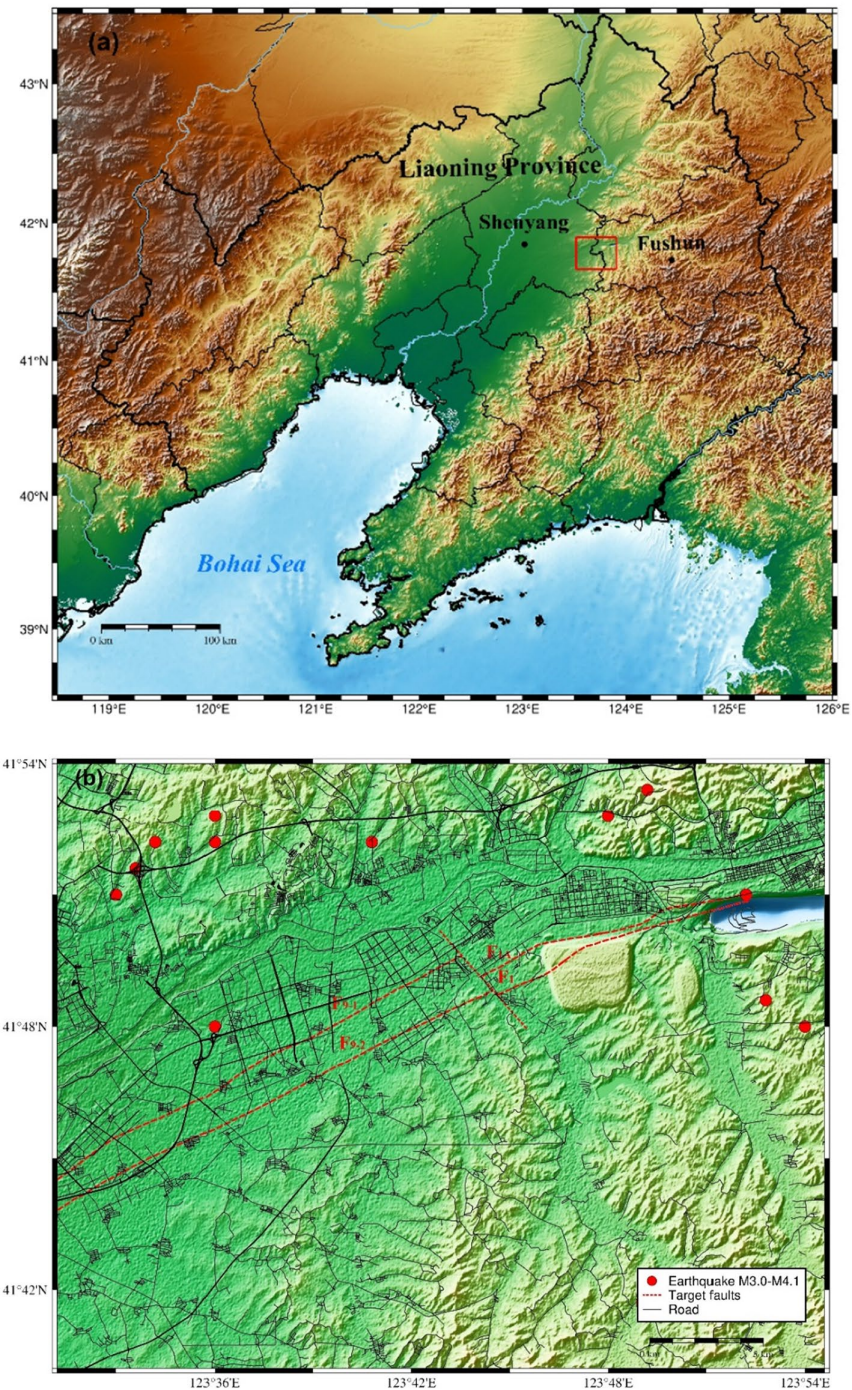

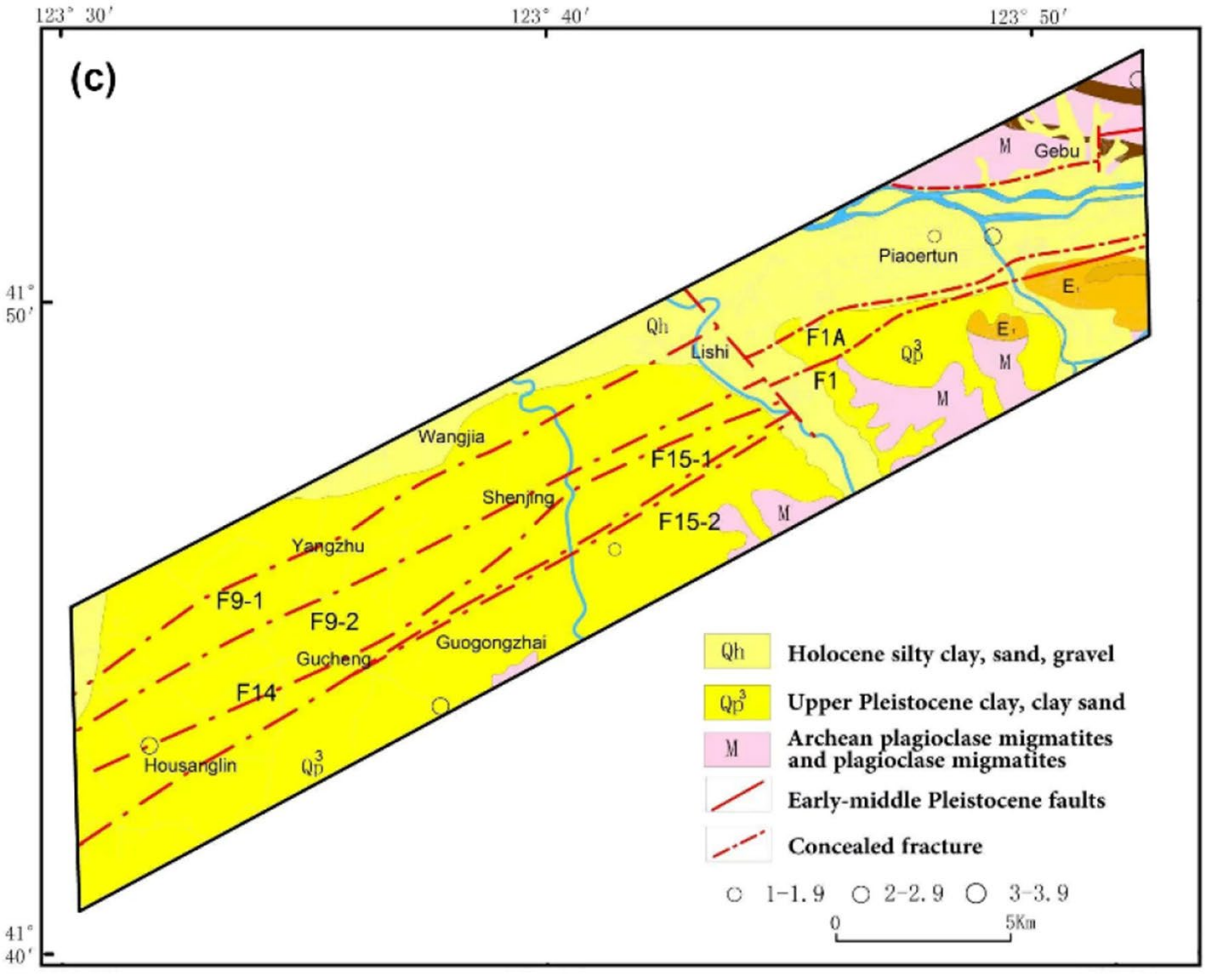
(**a**) Map of Liaoning Province, where the target area is located between the cities of Shenyang and Fushun (red rectangular box). The map was generated using the Generic Mapping Tools (GMT) (version 6.5) ^9^. (**b**) Terrain map of the study Shen-Fu New District. The red dotted line marks the traces of the target faults on the ground, while the surrounding residential areas and roads are marked in black. The distribution of historical earthquakes with magnitudes greater than 3.0 since 1970 is depicted by red circles (data are from the National Earthquake Data Centre ^10^). (**c**) Map of the seismic tectonics of the target area.

The study area is situated between 123.52° and 123.91° east longitude and 41.67° and 41.90° north latitude. It covers an area of approximately 1026 km^2^, with a rectangular shape measuring approximately 38 km east–west and 27 km north–south. The Hunhe Fault, which traverses this area, has an approximate length of 25 to 30 km. The two major ruptures causing seismic risk in the target area are F9 and F1. The two faults strike to the north-east-east and have a tendency towards the north–north-west with steep dip angles. Figure 1 illustrates the location and seismic tectonics of the target area.

**Figure 2:**
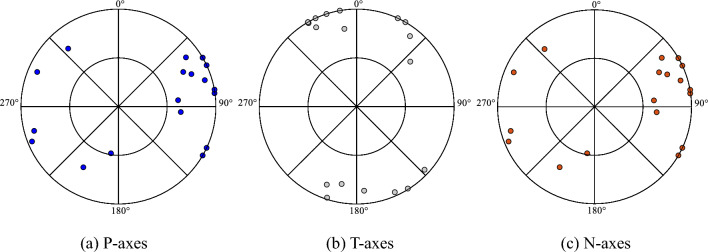
Spatial characteristics of the tectonic stress axis.

The tectonic stress field is the fundamental driver of rupture tectonic activity and seismicity. To investigate the characteristics of the tectonic stress field, the source mechanisms of historical seismic events were analysed over a larger range (122.25° to 124.57°E longitude and 40.97° to 42.25° N latitude) covering the target area. According to references^11–13^, in this area, 19 primary earthquakes with relatively complete seismic phase data occurred between 1974 and 1999 and between 2008 and 2016. Among these earthquakes, one had a magnitude of 2–3, thirteen had a magnitude of 3–4, two had a magnitude of 4–5, and three had a magnitude of 5–6. With the focal mechanisms of these events, the P-axis and T-axis are projected onto the Wulff net according to the azimuth and dip angles, revealing a concentrated distribution of points (Fig. 2). Of the P-axis orientations, 73% fall within the range of 54°-95° or 254°-293°. These results indicate that the principal compressive stress has a dominant north-east-east direction. Moreover, 89% of the T-axis direction falls within the range of 148°-201° or 330°-347°, indicating a dominant north–north-west direction for the principal tensive stress. Based on the dip angle analysis, dip angles less than 45° account for 84% and 100%, of the P and T stress axes, respectively; this result indicates that horizontal forces are major factors affecting the fault planes in the study area. Furthermore, the N-axes are also projected onto the Wulff net. An inclination greater than 45° accounts for 68%, corresponding to the horizontal directions observed in both the P and T axes. Thus, strike-slip faults are the predominant type of fault in the study area.

Regarding the Hunhe Fault in the Shen-Fu New District, the most recent activity since the Quaternary period has been characterised mainly by both normal dip-slip faults and right-lateral strike-slip movements. Furthermore, the southern and northern principal faults are inclined at steep angles towards each other, forming a graben basin. In summary, the distribution of the fault activity and tectonic stress conditions is generally conducive to the accumulation of ground stress. However, due to the low level of ground stress, the seismogenic ability of the fault is relatively limited. The most recent activity age of the fault segment is from the middle Pleistocene to the Quaternary.

A review of historical records indicates that more than 10 earthquakes of magnitude 3.5 or above have occurred near the Shen-Fu New District since 1518. The largest of these events was a magnitude 5.5 earthquake that occurred 10 km to the west of the target area in 1765, and the most recent earthquake in the target area was a magnitude 4.1 earthquake in 2003. Furthermore, an increase in the minor earthquakes has recently been observed in the area; however, earthquakes measuring 6 or above have not been documented. The target section of the Hunhe Fault can be identified as a rupture section with the characteristics of medium-strength seismic activity. The preceding analysis indicates that the Hunhe Fault zone in the study area has a seismic cycle of approximately 300 years. Over the next 50 to 100 years, the fault will undergo residual strain release and strain accumulation, increasing the risk of a potential earthquake with a maximum magnitude of 6.

## Earthquake hazard prediction method and model

### Hybrid technique for strong motion simulation

In this study, a hybrid technique combining a theoretical method and a semiempirical method is employed to simulate strong ground motion for the fault model. Previous studies^14–18^ have demonstrated that low-frequency ground motion can be simulated using deterministic source models and velocity structure models. However, high-frequency ground motion is inherently random due to the effects of source rupture and the small-scale tectonic complexity of the medium.

The propagation process of long-period seismic waves is simulated using the three-dimensional finite difference (3DFD) method. This method discretises the partial differential equation controlling the system’s motion state directly into spatial and temporal difference equations using mathematical meshes. In essence, the underground structure is assigned to the model grid. By applying an external force to the mesh of the seismic source portion, the induced motion is calculated grid by grid until the whole model is completed. This method employs an effective staggered-grid technique, as described in previous studies^19,20^. Furthermore, since these structures may significantly impact the propagation of seismic waves from the source to urban areas, a three-dimensional velocity model that accurately represents the irregular geological structures is needed. The propagation of waves in a three-dimensional, isotropic, linear elastic medium can be described by the velocity and stress components in the Cartesian coordinate system, as follows:

1 $$\rho \frac{{\partial v_{i} }}{\partial t} = \mathop \sum \limits_{j = 1}^{3} \frac{{\partial \sigma_{ij} }}{{\partial x_{j} }} + f_{i}$$

where $\rho$ represents the density of the medium in the epicentre area, $v_{i}$ represents the particle velocity of the elastic motion in the *i*th component, $\sigma_{ij}$ represents the *i*,*j*th component of the stress tensor, and $f_{i}$ represents the body force component.

On the other hand, high-frequency ground motion is synthesised using random vibration theory. Due to the lack of appropriate observation data, synthetic small event motions are simulated as semiempirical Green’s functions through the implementation of the stochastic method^21–23^; this method models high-frequency ground motions using white Gaussian noise with a finite duration. The fault plane of the target earthquake is divided into ${n}_{l}\times {n}_{w}$ elements, where ${n}_{l}$ and ${n}_{w}$ are the number of subevents along the length and width of the target fault, respectively. The strong motions from each subevent (${a}_{ij}$) are calculated by the stochastic point-source method and summed using appropriate time intervals corresponding to the duration of rupture propagation to obtain the ground motion acceleration of the entire fault $a\left( t \right)$.

2 $$a\left( t \right) = \mathop \sum \limits_{i = 1}^{nl} \mathop \sum \limits_{j = 1}^{nw} a_{ij} \left( {t + \Delta t_{ij} } \right)$$

where $\Delta t_{ij}$ is the relative delay time for the radiated wave from the *ij*th subfault to reach the observation point.

The synthetic amplitude spectrum consists of the source, path, site amplification factors, source amplitude spectrum, and site response. The site response is a frequency-dependent function that requires detailed data on the seismic profile of the target area.

Subsequently, a pair of matching filters is applied to eliminate the high-frequency content (above 1 Hz) from the 3DFD motion and low-frequency content (below 1 Hz) from the stochastically simulated motion. The filtered low-frequency seismic wave and high-frequency seismic wave are then superimposed and combined in the frequency domain, forming the broadband ground vibration time series of the same field via Fourier transform. In addition, the spatial distribution characteristics of the near-fault peak ground motions can be analysed. This hybrid technique combines deterministic and empirical approaches and has been demonstrated to enhance the efficiency of broadband strong ground motion simulation^24^; this technique is suitable for this study.

### Source model

In this study, the source kinematics model^25,26^ is employed to simulate the strong ground motion in the target area. The global source parameters comprise six independent parameters that describe the geometric characteristics and rupture types. These parameters are the strike angle, inclination angle, slip angle, length and width of the rectangular fault, and depth of the fault at the upper boundary. Furthermore, the global source parameters need to include the magnitude, the average slip or average stress drop on the entire fault, and the rupture propagation velocity. In contrast, local source parameters primarily affect and control the high-frequency components of strong ground motion at the source and near the fault, with secondary and noncontrolling effects on the low-frequency components. These parameters are closely connected to the heterogeneity or roughness of the fault, which consists of the number and location of asperities, the rupture initiation point and direction.

The dimensions of the faults were calculated based on the maximum magnitude of the potential earthquake, the fracture distribution pattern, and the geological occurrence. Consequently, both faults were defined as having a length of 15 km and a width of 10 km. Furthermore, the strike, dip and rake angles were determined^27^. The seismic depths of faults F9 and F1 were determined by referencing the information of the target area; this information included the deep velocity structure, deep electromagnetic characteristics, and precise positioning of small earthquakes. On a large scale, the earthquakes along the Tanlu Fault zone occurred mainly at depths ranging from 6 to 25 km. In the region in close proximity, the most recent seismic events had average depths of 8 to 12 km.

Somerville et al.^25^ defined asperities as regions on a fault that exhibit a high degree of slip relative to the average slip of the rupture area. The analysis of the characteristics of slip models for fifteen crustal earthquakes revealed that asperities accounted for approximately 22% of the total rupture area on average and represented 44% (equivalent to two times 22%) of the total slip on the fault. Moreover, the average number of asperities was determined to be 2.6. Consequently, in this study, both F9 and F1 were characterised as consisting of two asperities, designated A_sp-1_ and A_sp-2_. The seismic moment $M_{0}$ was calculated based on the maximum potential magnitude $M$, as specified in Eq. (3)^28^.

3 $$M = \frac{2}{3}{\text{log}}M_{0} - 10.7$$

In addition, the areas of the largest asperity A_sp-1_ and secondary asperity A_sp-2_, as well as the rise time $T_{R}$, were determined in accordance with Eqs. (4) to (6) ^25^.

4 $${\text{A}}_{{{\text{sp}} - {1}}} = 3.64 \times 10^{ - 16} \times M_{0}^{2/3}$$

5 $${\text{A}}_{{{\text{sp}} - {2}}} = \left( {5 - 3.64} \right) \times 10^{ - 16} \times M_{0}^{2/3}$$

6 $$T_{R} = 2.03 \times 10^{ - 9} \times M_{0}^{1/3}$$

The rupture propagated in a concentric radial pattern along the faults, with a velocity of 2.7 km/s, and the average asperity slip contrast was set at 2.01^25^. Subsequently, the seismic moment and stress drop on the largest and secondary asperities, as well as the background, could be obtained. Based on the above analysis, the seismic source parameters of F9 and F1 are listed in Table 1.

**Table 1:** Seismic source parameters for the magnitude 6.0 earthquake on the F9 and F1 branches of the Hunhe Fault zone.

Parameter	F9	F1
Fault length (km)	15	15
Fault width (km)	10	10
Strike (°)	245 ~ 247	250 ~ 255
Dip (°)	55 ~ 65	50 ~ 70
Rake (°)	-120 ~ -135	-120 ~ -135
Upper depth of fault (km)	5	6
Lower depth of fault (km)	14	15
Length-wise segmentation	15	15
Width-wise segmentation	10	10
Fault element length (km)	1	1
Fault element width (km)	1	1
Sample frequency (Hz)	100	100
Fault area (km^2^)	150	150
Seismic moment (dyne·cm)	1.12 × 10^25^	1.12 × 10^25^
Average seismic moment of fault elements (dyne·cm)	0.6 × 10^22^	0.6 × 10^22^
Largest asperity area (km^2^)	18	18
Secondary asperity area (km^2^)	8	8
Rise time (s)	0.45	0.45
Rupture velocity (km/s)	2.7	2.7
Average asperity slip contrast	2.01	2.01
Seismic moment on the largest asperity (dyne·cm)	0.3 × 10^25^	0.3 × 10^25^
Seismic moment on the secondary asperity (dyne·cm)	0.1 × 10^25^	0.1 × 10^25^
Seismic moment on the background (dyne·cm)	0.72 × 10^25^	0.72 × 10^25^
Average stress drop (bar)	14.9	14.9
Stress drop on the largest asperity (bar)	95.7	95.7
Stress drop on the secondary asperity (bar)	107.7	107.7
Stress drop on the background (bar)	12.7	12.7

In general, the impact of asperities on the prediction of strong ground motion is considerable and directly influences the prediction of strong ground motion. In reality, the asperities are situated at the deep level of the fault, and thus, previous studies of asperities have predominantly involved the inversion of existing earthquakes, and the majority have focused on the physical mechanism, geometric characteristics, and motion process during earthquakes, which are inherently uncertain due to the inaccessibility of the underground. In this study, the approach is to establish an asperity source model for earthquakes based on the principle of adverse or average conditions for the target area, considering the inherent uncertainty of different source scenarios. As the F9 and F1 faults structurally intersect the north–south faults in the target area, the asperities were set according to the intersections as boundaries. F9 is situated within the core of the target area and has a more pronounced impact on the target area, two scenarios were set up considering the fault scale. Scenario 1 was selected because it likely had the most detrimental impact on the target area. In contrast, in the southern section, reference scenario 2 was used to elucidate the uncertainty of the set parameters with spatial variations in the primary and secondary asperities. Since F1 is situated in the north-east of the target area and has a relatively limited impact on the core area, a scenario was constructed that represented a relatively adverse situation.

**Figure 3:**
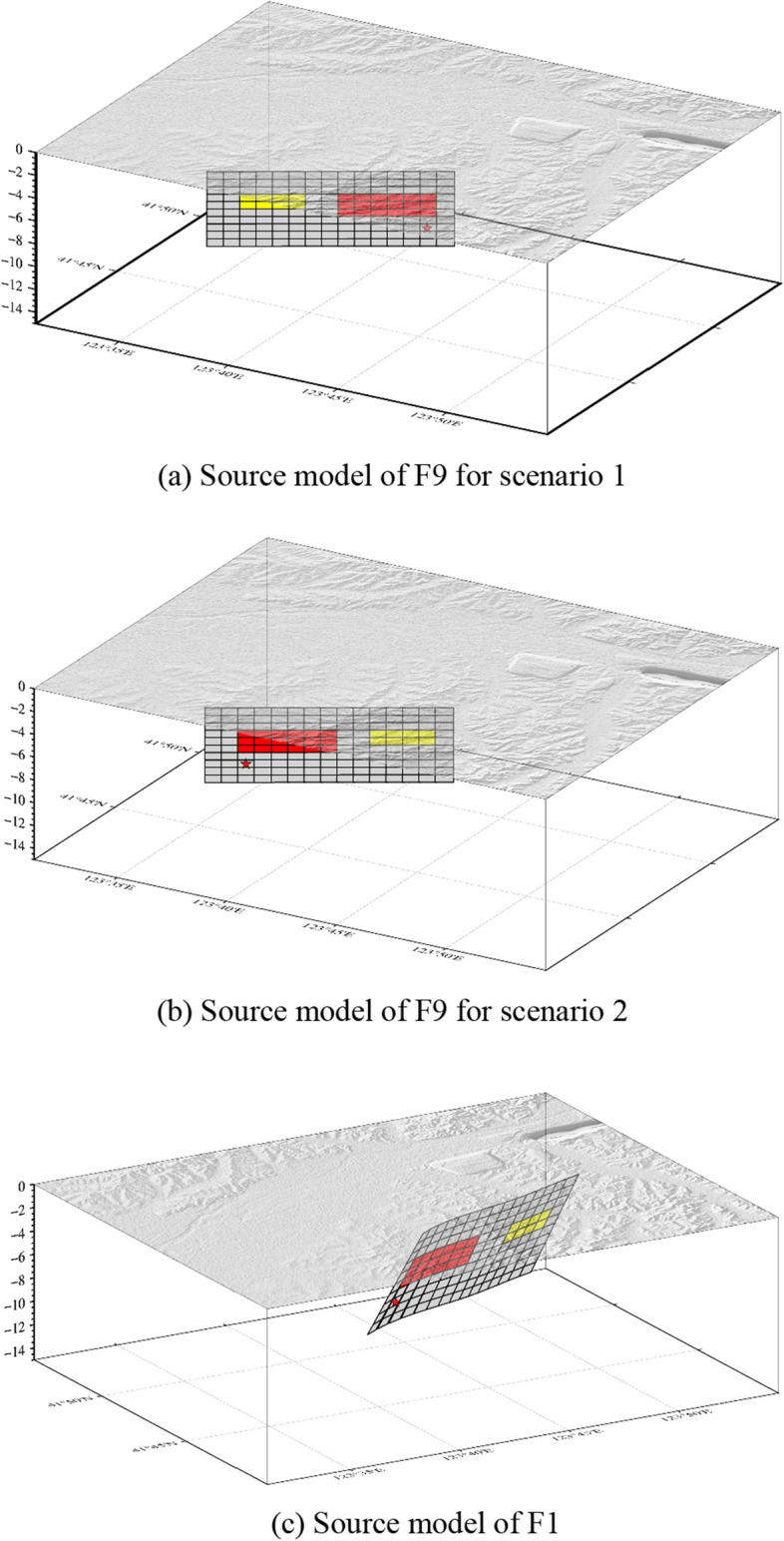
Characterised source model of F9 and F1; rectangular regions with colours show the area of the asperities, red for A_sp-2_ and yellow for A_sp-2_. The star symbol indicates the rupture starting point.

Regarding the starting point, the Hunhe Fault zone consists of numerous branch fault patterns intersected by north–south faults. Due to this configuration, the stress is concentrated at these intersections, increasing the likelihood of earthquakes. Furthermore, the starting point of a rupture is assumed to be located at the lower end of the primary asperity to consider the near-fault effects, such as the front rupture effect, and to simulate the seismic source that is dangerous to the target area. The characterised source models of F9 and F1 are shown in Fig. 3.

### Velocity structure model

**Figure 4:**
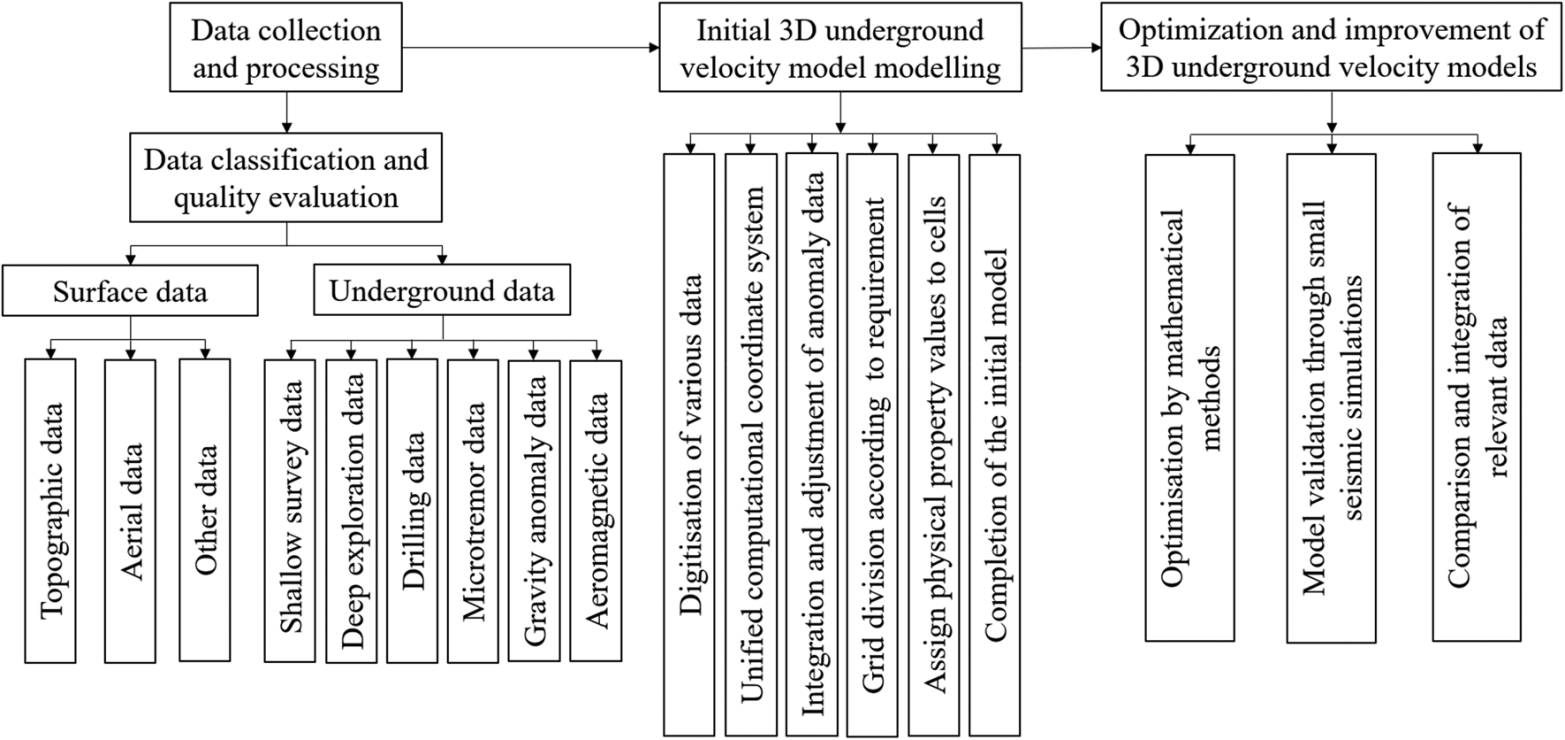
Flowchart of the velocity structure modelling process.

The sediments filling the basins could amplify ground motions, and their velocity structures complicate the propagation of seismic waves^29,30^. Therefore, the three-dimensional velocity structures of these urban basins need to be determined to predict the strong ground motions. Figure 4 illustrates the flowchart for the modelling process.

The target area is characterised by complex geological and tectonic conditions and is situated in the transitional tectonic zone between the Jiaoliao terrace and the Lower Liaohe fault at the edge of the Sino-Korean Para-platform. The Quaternary basins in the area are well developed, with the thickness of deposits rapidly increasing from east to west, ranging from approximately 20 m to approximately 100 m.

**Figure 5:**
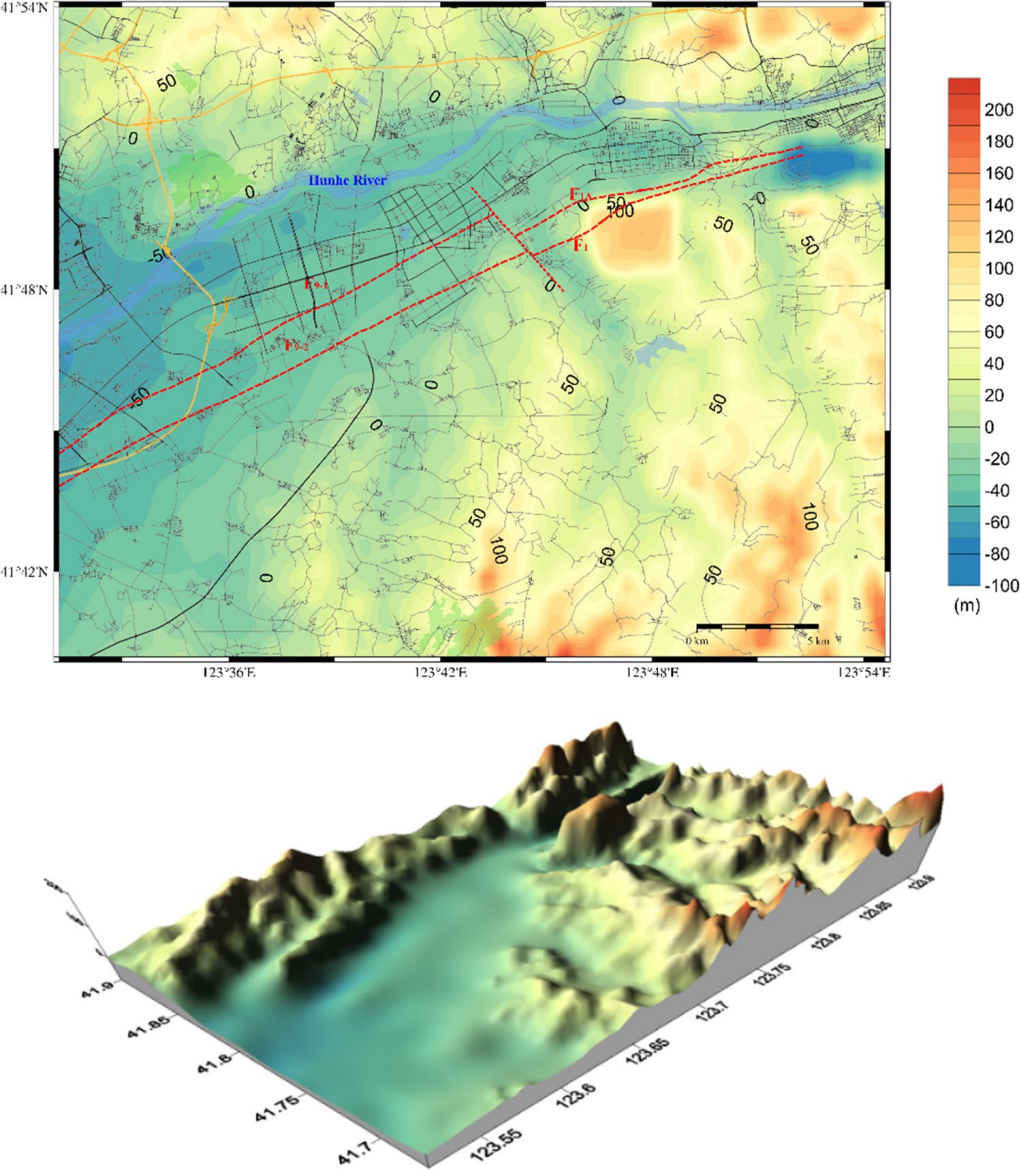
Contour map of the velocity structure model of the corresponding Q_p1_ layer.

The distribution and physical parameters of the various strata of the target area were determined simultaneously and sequentially. This was achieved by utilising the results from shallow seismic exploration, geological and topographical data, borehole logging data, and wave velocity data of different strata, as well as microtremor surveys. Furthermore, the three-dimensional velocity structure model was divided into seven layers. The Quaternary stratigraphy is classified into four layers: Q_h_, Q_p3_, Q_p2_, and Q_p1_; the Q_p1_ layer forms the base of the Quaternary system. The N layer corresponds to the tertiary system, the R layer represents the lithosphere layer, and the Moho layer represents the seismogenic zone. Each layer is composed of seven physical parameters (as detailed in Table 2); these include the spatial coordinates (*x*_j_,* y*_j_, *z*_j_) of the medium elements, P-wave velocity, S-wave velocity, density, and the quality factor* Q*. These parameters are primarily determined according to Liao et al.^31^ and Brocher^32^. Figure 5 provides an illustration of a velocity structure model for the Q_p1_ layer as an example.

**Table 2:** Physical parameters of the velocity structure model in the study area.

Layer	P-wave velocity (km/s)	S-wave velocity (km/s)	Density (g/cm^3^)	*Q*
Q_h_	0.80–1.00	0.20–0.30	1.75–1.80	50–60
Q_p3_	1.20–1.50	0.30–0.50	1.85–1.90	70–80
Q_p2_	1.70–1.90	0.60–0.70	1.90–1.95	80–100
Q_p1_	2.00–2.20	0.80–0.90	2.00–2.05	120–150
N	3.80–4.80	2.40–2.60	2.50–2.60	300–350
R	5.40–6.00	2.80–3.00	2.70–2.80	500–700
Moho	6.20–7.00	3.20–3.50	2.80–2.90	> 1000

## Results from the predicted strong ground motion of the earthquake scenarios

**Figure 6:**
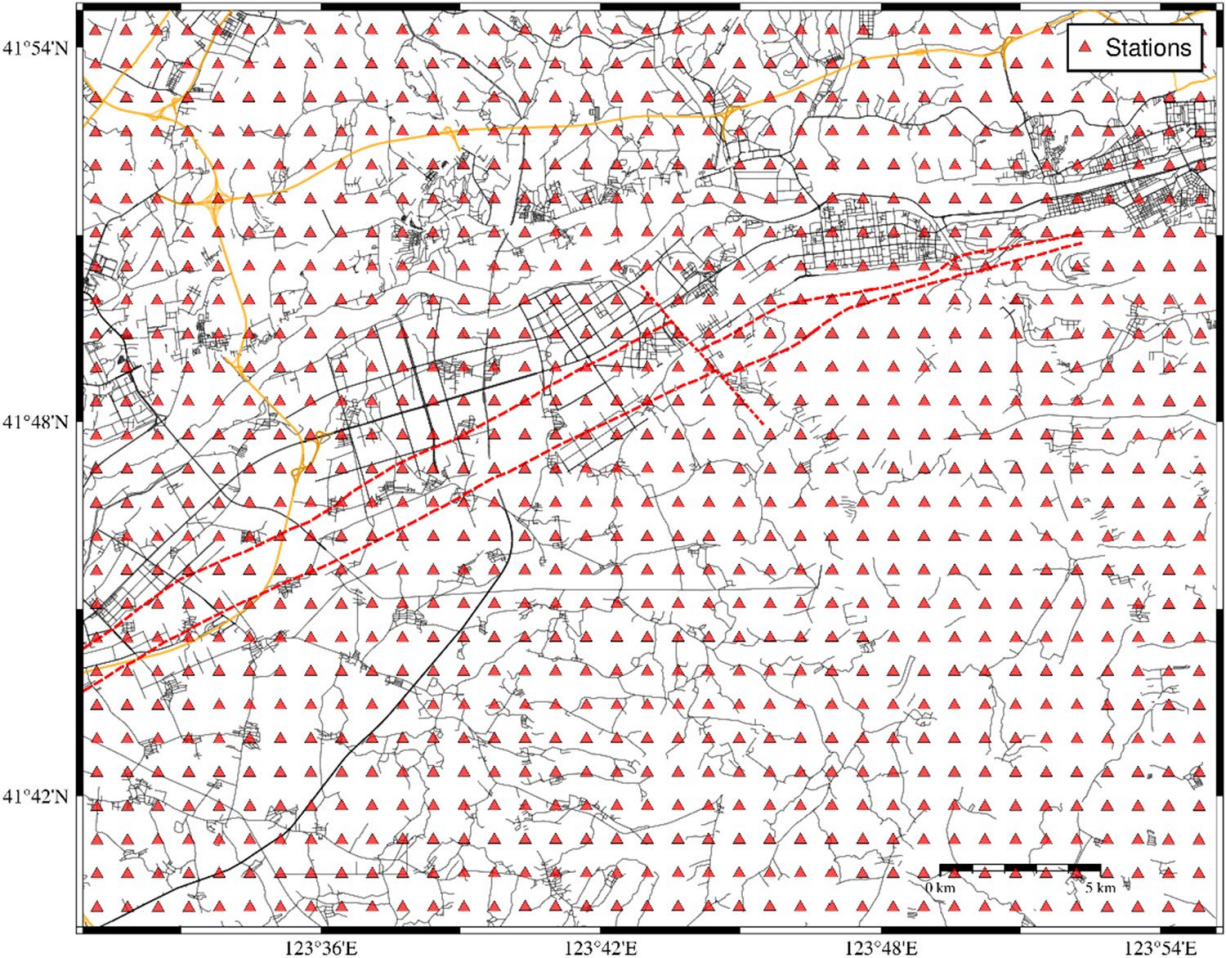
Plan view of the computational area, with the red triangles indicating the locations of the virtual stations on the surface. Each station is separated by an interval of 1 km.

Figure 6 shows a plan view of the computational area, which encompasses an area of approximately 1026 km^2^ with a length of approximately 38 km from east to west and a width of approximately 27 km from north to south. Seismic wave propagation in the range of 38 km × 27 km × 20 km was simulated with a model comprising 380 × 270 × 200 grid points, a grid size of 0.1 km, and a time step of 0.01 s. The red triangles indicate the locations of the virtual stations on the surface, with an interval of 1 km between each station. The ground motion, which includes the time series of acceleration, velocity, and displacement, was calculated for each point in three components: north–south, east–west, and up-down. The maximum values were extracted from all three components.

### Scenario of a 6.0 magnitude earthquake on the F9 branch fault

**Figure 7:**
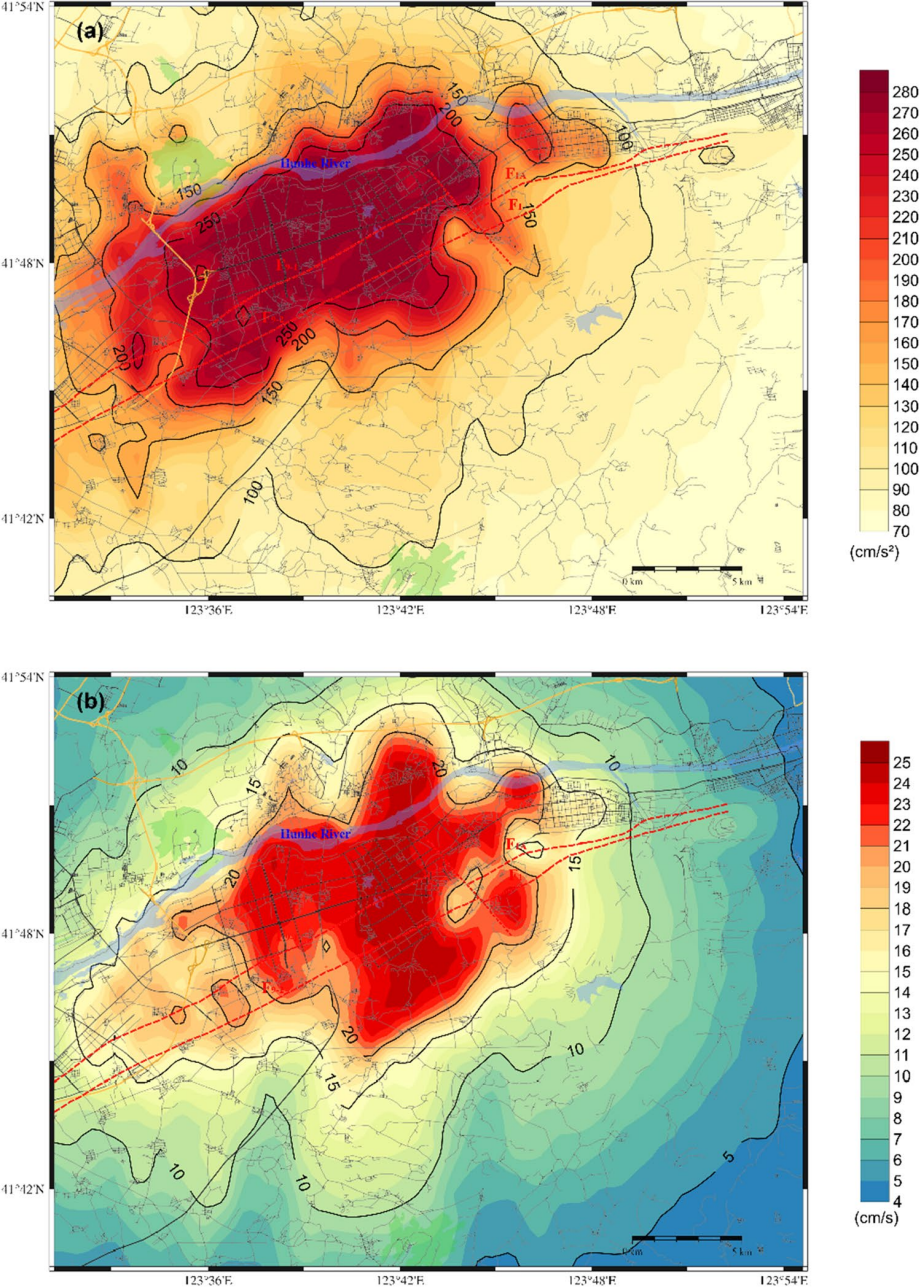

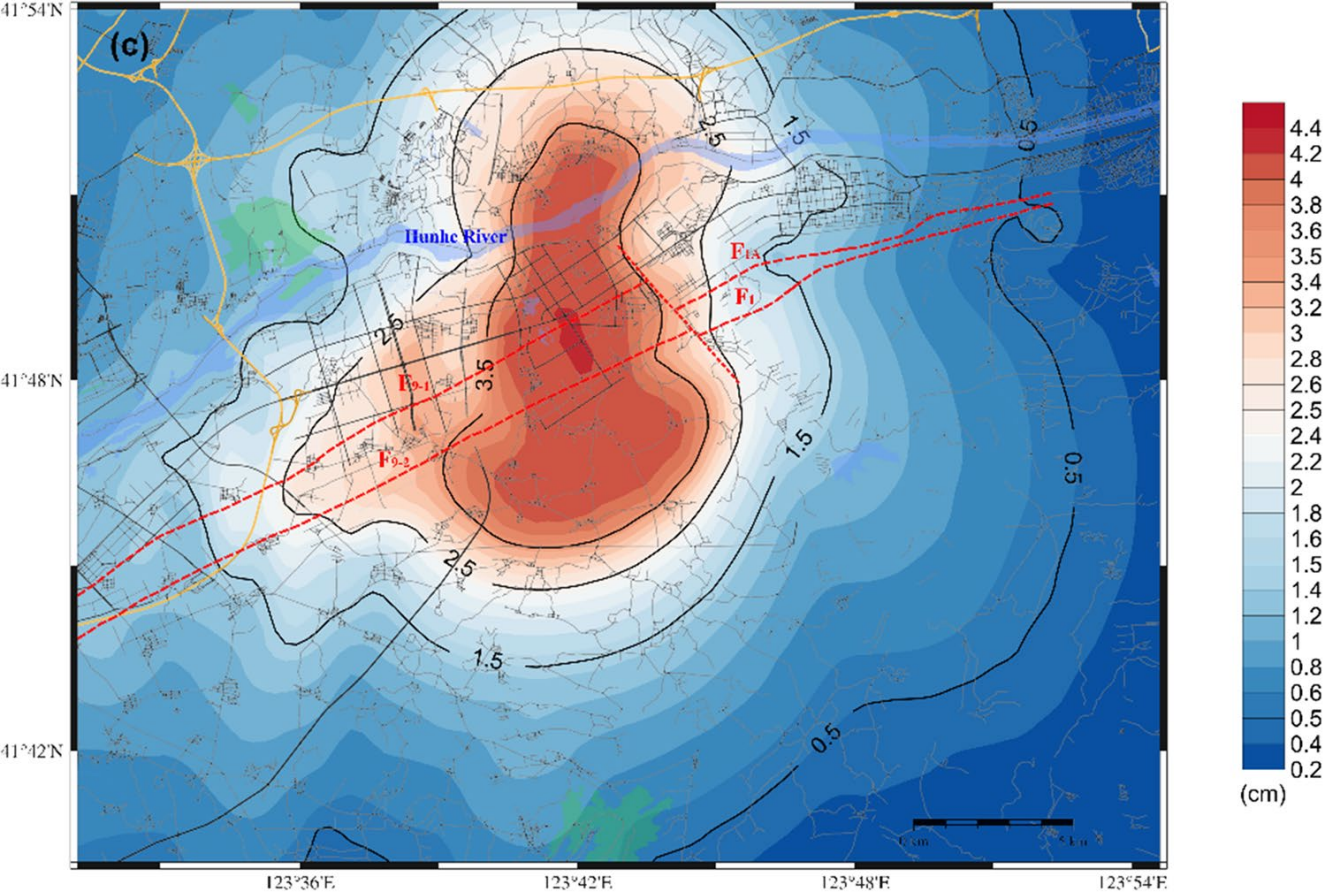
Distribution of the predicted ground motion of scenario 1 for a 6.0 magnitude earthquake on the F9 branch fault; (**a**) for the PGA; (**b**) for the PGV; (**c**) for the PGD.

Figure 7 shows the distributions of the predicted PGA, PGV, and PGD within the target area for the F9 branch fault under scenario 1.

As illustrated in Fig. 7(a), the entire target area is affected by ground motion, with the region on either side and at the ends of the fault experiencing a more significant impact. This results in an irregular oblong distribution with a long axis of 30–35 km and a short axis of 20–25 km, oriented in a northeast-southwest direction. The PGA in this area exceeds 100 cm/s^2^, indicating the potential for damage to structures in the core area of the Shen-Fu New District. The amplitude of ground motion close to the fault trace is estimated to be greater than 200 cm/s^2^, with the limiting value potentially exceeding 270 cm/s^2^. The ground motion in the Hunhe River Valley sedimentary area on the northwestern side of the fault is greater than that in the diluvial terrace and diluvial fan area on the southeastern side of the fault, where the estimated PGA is approximately 70–100 cm/s^2^.

The ground motion caused by the active faults in the target area exhibits source features and site effects. The seismic source impact consisted of the fault seismicity mechanism, spatial distribution, asperity location, and rupture starting point. Since the F9 branch features a normal fault with a right-lateral strike-slip rupture mode, the ground motion is relatively well developed along the fault trace and the hanging wall on the northwest side. Regarding the site conditions, the geological structure surrounding the fault mainly consists of hills, floodplains of the pre-mountain region and valley troughs, resulting in complex superposition effects on the ground motion.

Figure 7(b) and Fig. 7(c) illustrate that the distribution patterns of the PGV and PGD are analogous to that of the PGA, showing a relatively diminished influence area. The high-value impact zone exhibits a distribution aligned with the direction of fault spreading and is significantly influenced by the focal mechanism and the distribution of the loose deposits in the surficial geology, as well as the complex edge effect of the geological structure features. This results in the formation of a high-value ground motion field in the northwest-southeast direction perpendicular to the fault; here, the main high-value impact zone is notably concentrated on the northwest side of the fault. In the target area, the extreme PGV and PGD are approximately 25 cm/sec and 4.4 cm, respectively.

**Figure 8:**
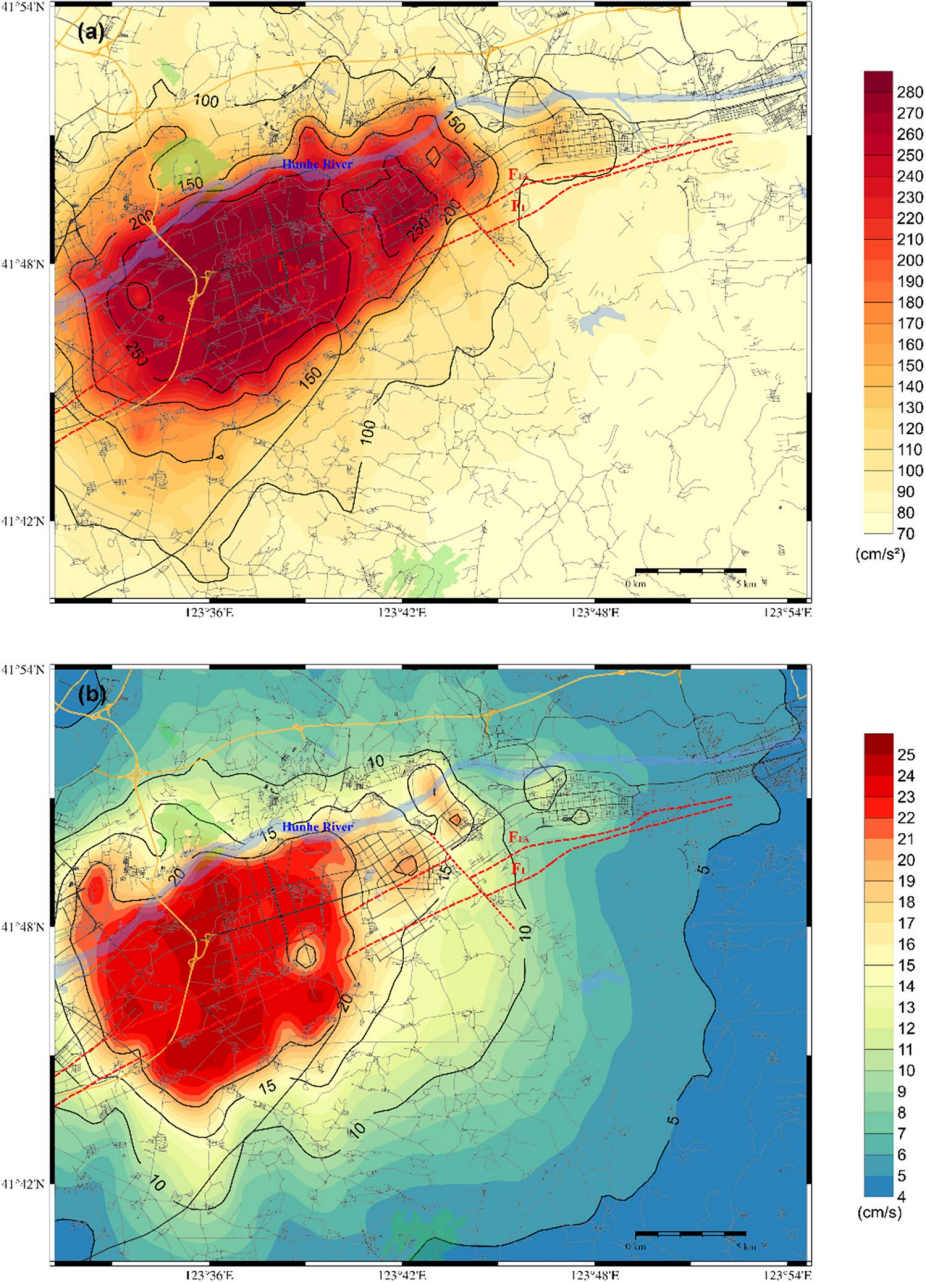

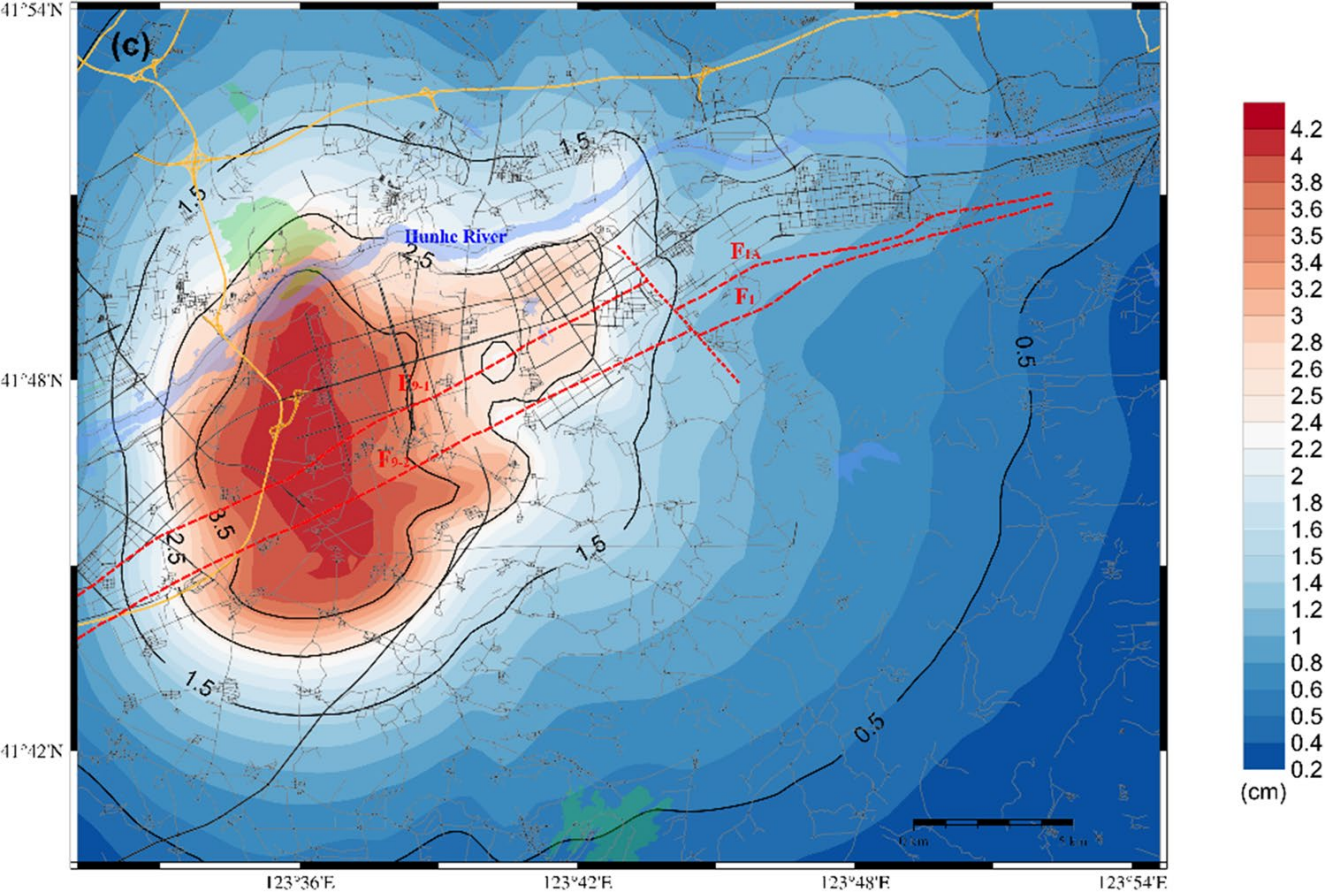
Distribution of the predicted ground motion of scenario 2 for a 6.0 magnitude earthquake on the F9 branch fault; (**a**) for the PGA; (**b**) for the PGV; (**c**) for the PGD.

Regarding scenario 2 of the F9 branch fault, the distributions of the PGA, PGV and PGD are shown in Fig. 8. A comparison of the two scenarios reveals that both scenarios have a significant impact on the core area of the Shen-Fu New Area. The former model exerts a more pronounced influence on the eastern urban area, while the latter exerts a more pronounced influence on the western urban area. The extreme PGA approaches 280 cm/s^2^, while the maximum PGV and PGD values are approximately 25 cm/sec and 4.2 cm, respectively. Furthermore, the distribution of ground motion is influenced by the characteristics of the seismic source and the site conditions, resulting in significant local inhomogeneity in the spatial distribution. The Hunhe River alluvial plain and the complex underground structure of the bordering areas of the plain and the hills exert complex amplification on the ground motion.

### Scenario of a 6.0 magnitude earthquake on the F1 branch fault

**Figure 9:**
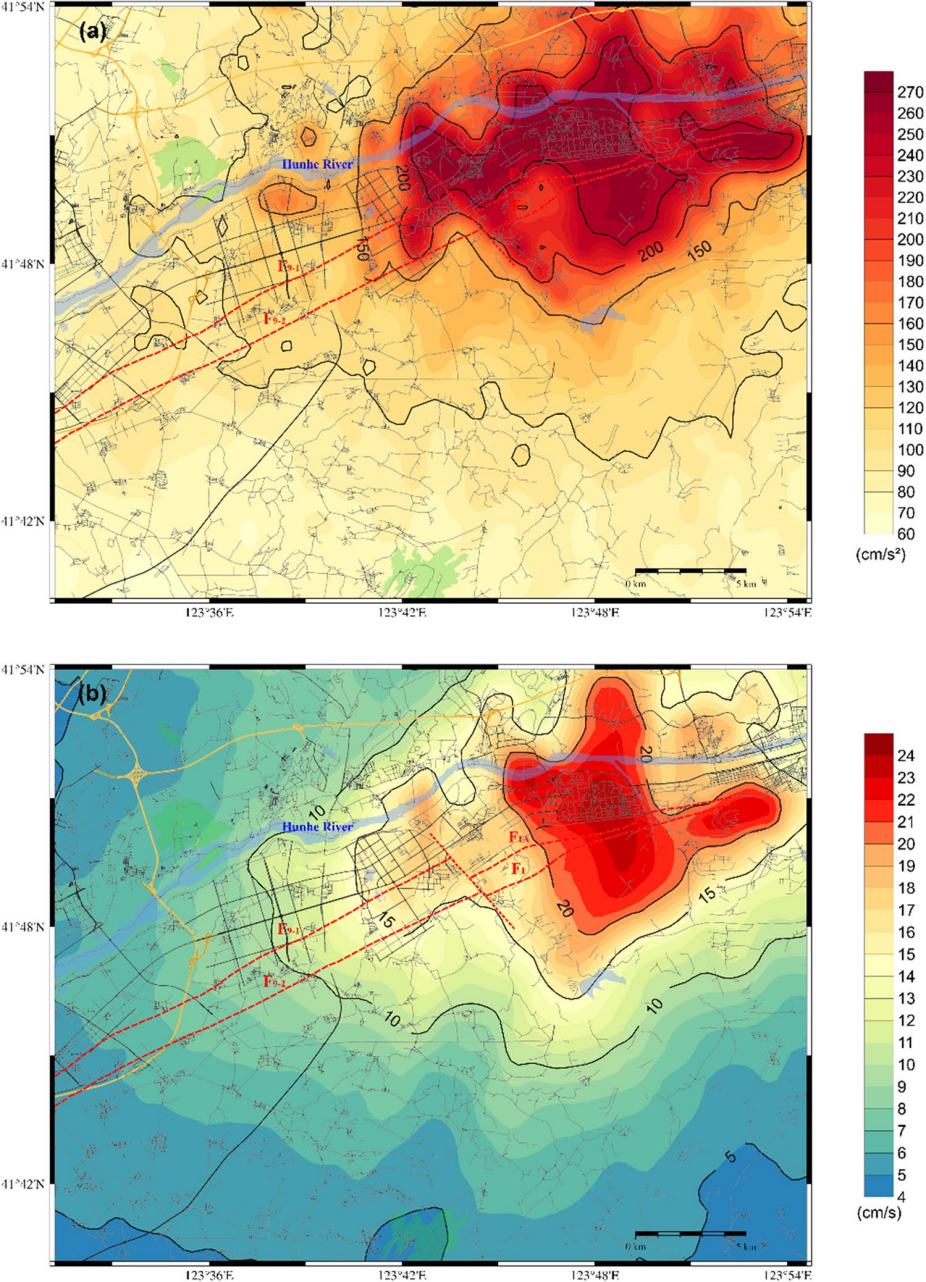

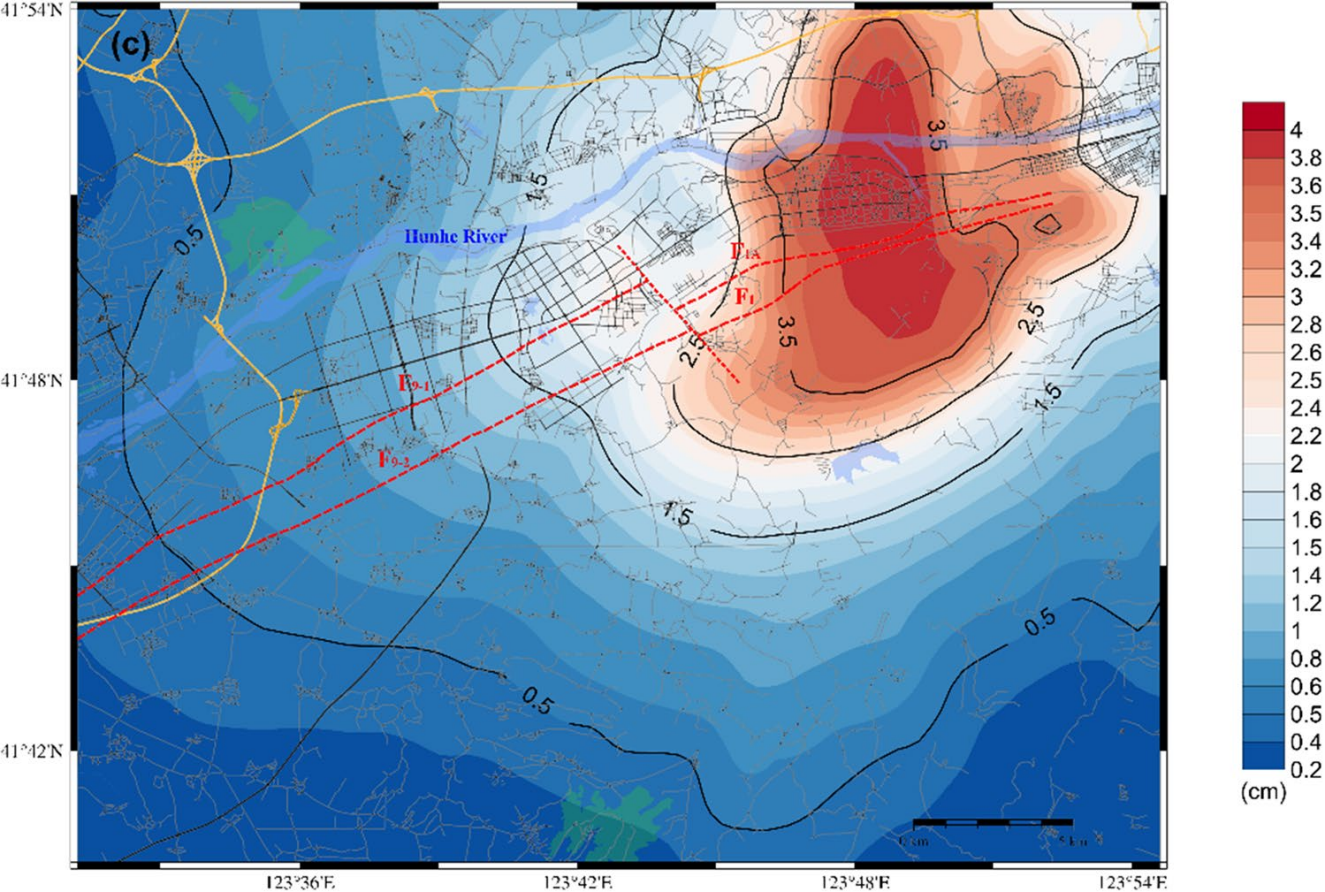
Distribution of the predicted ground motion of the scenario of a 6.0 magnitude earthquake on the F1 branch fault; (**a**) for the PGA; (**b**) for the PGV; (**c**) for the PGD.

Regarding the F1 branch fault, the distributions of the predicted PGA, PGV and PGD within the target area when the earthquake occurs are illustrated in Fig. 9. Figure 9(a) shows that the PGA distribution has a significant impact on the northeastern part of the core area, and the PGA reaches a value greater than 260 cm/s^2^. These strong ground motions originate from the ruptures in the source zones of the faults that are distributed in this area. Furthermore, a depression is located at the end of the F1 fault, and the ground motion is considerably elevated due to the influence of this terrain. The intensity of ground motion decreases as it moves from the east towards the west along the valley; here, in the western and southern parts of the target, the ground motion decreases substantially to approximately 60–100 cm/s^2^.

As shown in Fig. 9(b) and Fig. 9(c), the distribution patterns of PGV and PGD exhibit similarities to that of PGA, with a narrower impact range. The highest PGV and PGD values observed within the target area are approximately 24 cm/sec and 4 cm, respectively.

### Simulation comparison with the ground motion prediction equations

To ascertain the reliability of the simulation results, the characteristics of the PGA on the investigated fault are compared with those of suitable ground motion prediction equations (GMPEs); these GMPEs also represent a crucial component in the field of seismic hazard assessment. Currently, China is utilising the fifth generation of zoning maps released in 2015; these maps were developed using the GMPEs proposed by Yu et al.^33^. The model classified China into four distinct regions, and each region is characterised by its own seismic activity and ground motion attenuation characteristics. For each region, a set of GMPEs was developed for the long axis (along the direction of fault strike) and short axis (perpendicular to fault strike). The model is illustrated in Eq. (7):

7 $${\text{log(}}Y) = A + BM + C{\text{lg}}\left( {R + De^{EM} } \right)$$

where Y is the peak acceleration $a_{E}$, M is the magnitude, R is the epicentral distance, and A, B, C, D and E are regression coefficients. For the regions where the target area is situated, the coefficients for earthquakes with magnitudes less than 6.5 are listed in Table 3, where σ is the standard deviation.

**Table 3:** Coefficients of the attenuation relation of ${a}_{E}$ for study regions with magnitudes less than 6.0.

${a}_{E}$	A	B	C	D	E	σ
Long axis	2.417	0.498	-2.079	2.802	0.295	0.236
Short-axis	1.715	0.471	-1.723	1.295	0.331	0.236

**Figure 10:**
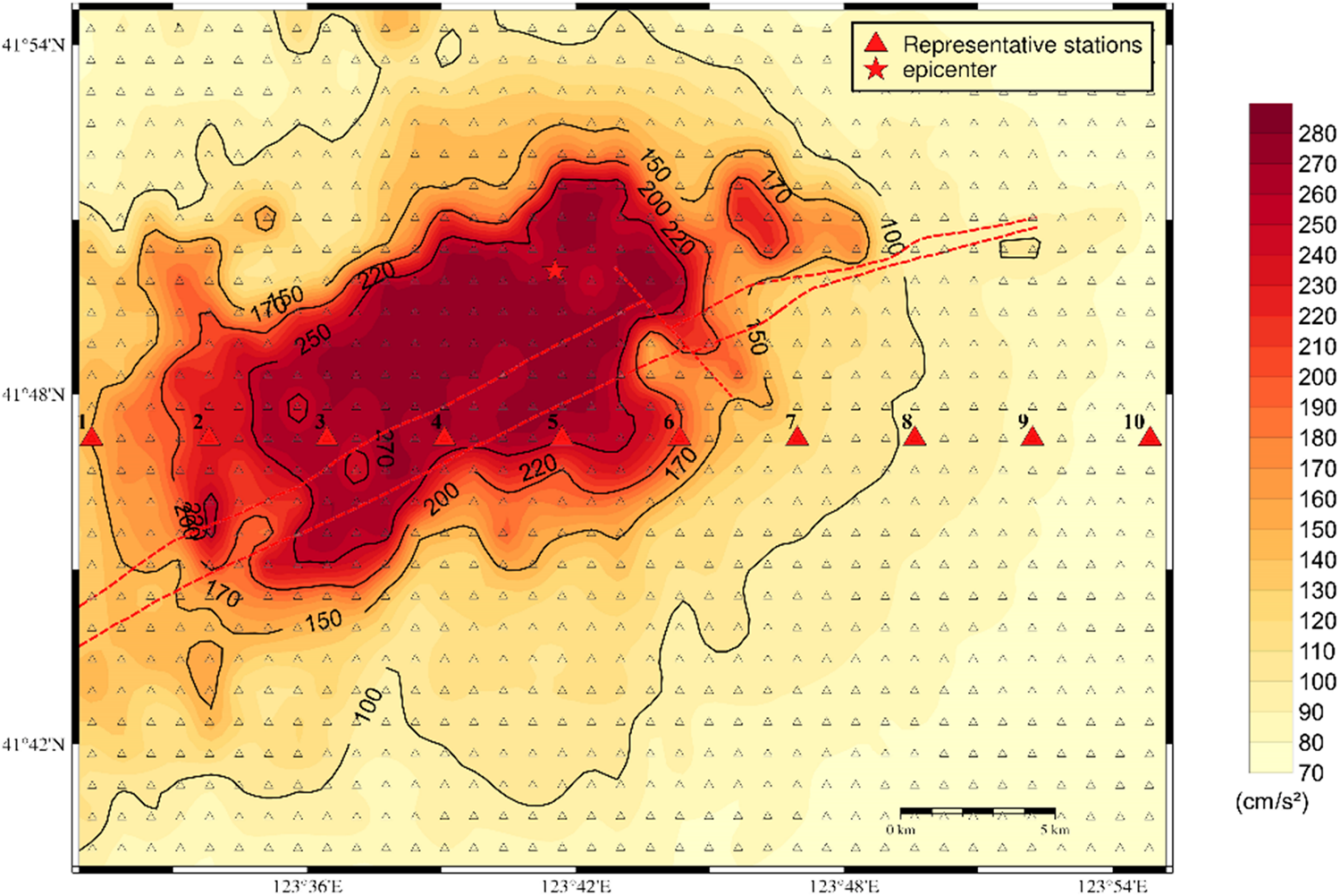
Distribution of the ten selected representative stations of scenario 1 for the F9 branch fault.

**Figure 11:**
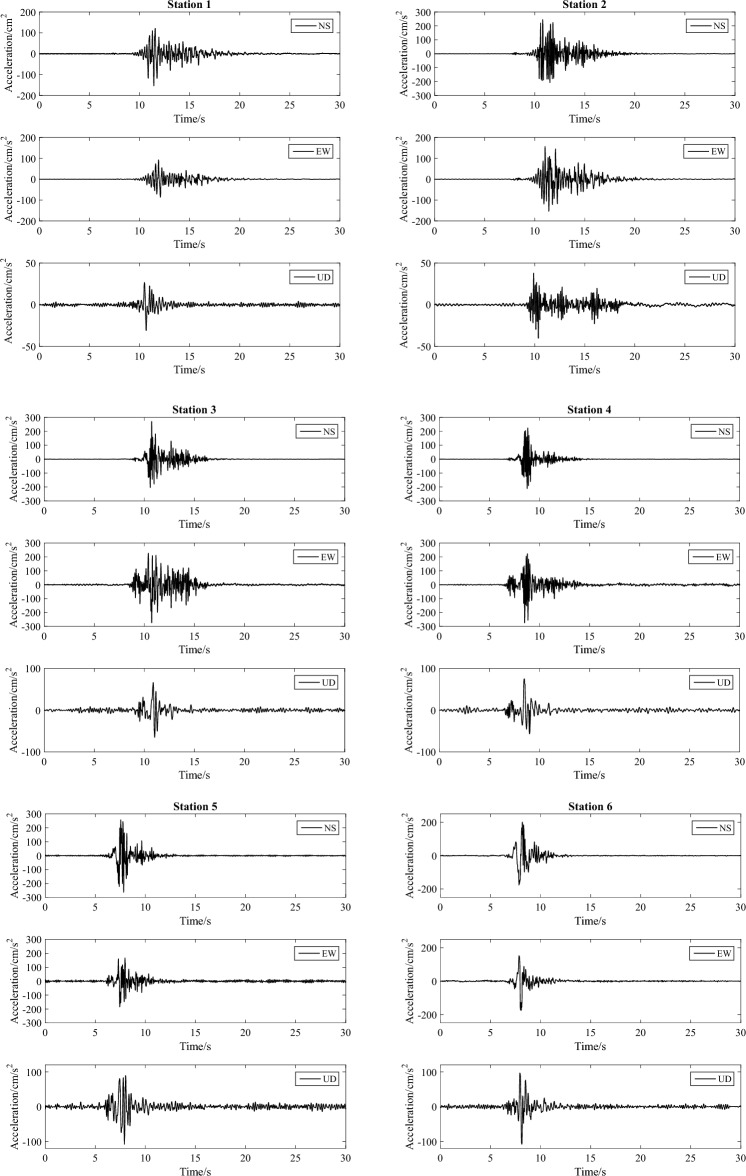

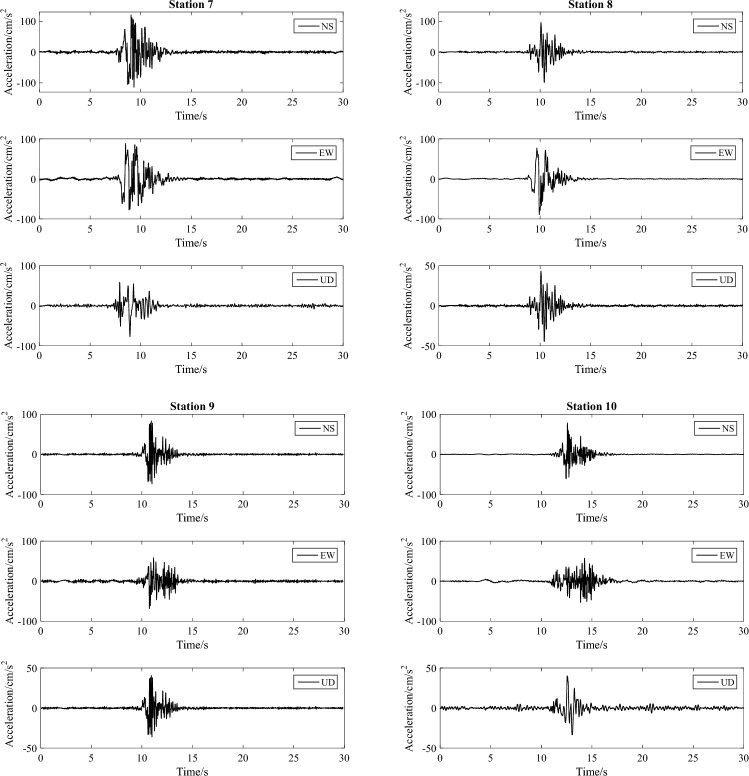
Corresponding three-component waveforms of the ten selected representative stations.

**Figure 12:**
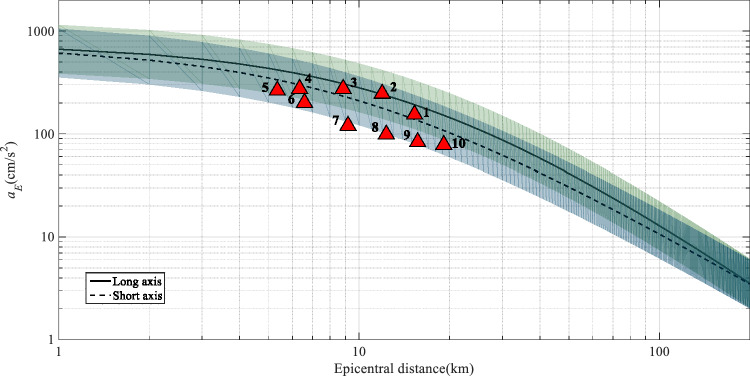
Median curves of the PGA as a function of epicentre distance for the study regions in China. The solid lines represent the long-axis direction, and the dashed lines represent the short-axis direction. The shading indicates the standard deviation. The ten triangles indicate the representative virtual stations.

As illustrated in Fig. 10, we selected ten representative stations of scenario 1 of the F9 branch fault as examples. The corresponding three-component waveforms are depicted in Fig. 11. Figure 12 shows the PGA of each station versus its epicentral distance, with the GMPE curves superimposed.

In general, the PGA of the earthquake scenario correlates well with the attenuation relationship, and the results are represented on the curve or within its standard deviation. Specifically, due to the hanging wall effect, the PGAs of stations 1–4, which are located in the hanging wall, are greater than those of stations 5–10, which are located in the footwall. Furthermore, the ground motion in the Hunhe River valley on the northwestern side of the fault is considerable; however, in the area of the diluvial terrace and diluvial fan on the southeastern side of the fault, the ground motion is relatively moderate. This evidence indicates that the simulated ground motion can respond to the characteristics of the fault and the site conditions.

## Discussion

One of the primary factors that impacts the PGA distribution is the focal mechanism of the earthquake; this mechanism includes the fault geometry and rupture features. Another significant factor is the regional geological structure. Furthermore, the target faults reach 15 km in length, and the fault projection area is extensive due to the dip angle, resulting in a wide range of influence. Furthermore, the thick deposits in the target area have a significant amplification effect on ground motion, as demonstrated by  Herrero-Barbero et al.^34^. In addition, strong accelerations are observed in the direction perpendicular to the faults, as well as in the western segment of the faults. Considering the fault mechanism, initially, the faults are predominantly normal faults with a right-lateral strike-slip rupture mode, and the ground motion close to the fault traces displays fluctuations in the hanging wall effect, slip effect, velocity pulse effect, and vertical effect. The initial magnitudes of the impacts are less pronounced than those of the dip-slip reverse fault, although the vertical impact is more significant. Moreover, the vertical effect of strong ground motion close to the fault tends to increase with increasing dip angle. Second, the fault rupture mode generally follows the fault strike, leading to a region of high ground motion on the surface due to a large-amplitude pulse waveform that forms in front of the fault rupture. This impact has been validated in the analysis of data from the Chi-Chi earthquake^35,36^.

The distribution of PGV indicates that areas with high velocity values are predominantly concentrated near faults. The mechanism responsible for the high-value region is fundamentally consistent with that previously analysed in relation to the PGA. The area experiencing high velocity corresponds mainly to the thickness of the loose cover layer; this indicates that the velocity distribution is sensitive to the subsurface structure. In summary, the distribution of the PGV is relatively uniform, which aligns with the trends from the spectral composition of the long-period wave in the velocity. The distribution of the PGD reveals pronounced variations, which are strongly correlated with the location, length, and width of the surface rupture or strong deformation zone. A high-value zone is apparent in the central region of the northern side of the fault, which reflects the fault mechanism. The displacement of the surface, including the strong deformation zone, is most significant along the fault trace and is an important characteristic of active faults. Thus, despite the damage caused by acceleration, the surface ruptures or deformations can likely result in significant deformation disasters for buildings. Consequently, this result has great significance for the seismic defence of new buildings.

The earthquake prediction method employed in this study considers various factors, including source characteristics, propagation path, and site effects. The fundamental principle of this approach is to identify and determine the largest possible earthquake that may occur, and then the deterministic method is used to provide detailed ground motion predictions. This approach differs from the technique employed for setting the seismic design ground motion in the current seismic code, as evidenced by the “Seismic ground motion parameter zonation map of China”^37^ and seismic safety evaluation. The basic premise of the current seismic code is to provide an estimation of the mean ground motion for a city or site by forecasting the anticipated development trend of earthquakes over a future period (typically 50 or 100 years). This involves the application of probabilistic statistical techniques in conjunction with the assessment of seismic risk at the target location. The methodology for designing ground motion (mean and variance) is based on the assessment of the recurrence frequency of earthquakes and the exceedance probability required by the seismic design class. This study examines the seismic hazard of a potential 6.0 magnitude earthquake occurring in the Shen-Fu New District. Consequently, the ground motion level would exceed that of a major earthquake (with a return period greater than 2000 years), as outlined in the “Seismic ground motion parameter zonation map of China.” If the seismic design is carried out with the proposed ground motion system, the design goal can ensure that in the instance of a potential maximum earthquake, even if some buildings are damaged, a significant portion of urban facilities and houses will remain undamaged, thus accomplishing the aim of earthquake prevention and disaster reduction.

## Conclusion

In this paper, an analysis of the seismic risk and hazard associated with the Hunhe Fault in the Shen-Fu New District is presented. The target F9 and F1 faults have the potential to generate a magnitude 6.0 earthquake in the next 50 to 100 years. Subsequently, a hybrid technique was used to simulate the ground motion of the two faults, where the resulting distributions of the PGA, PGV, and PGD were reported and analysed. The seismic event under discussion is classified as a low probability earthquake, comparable to or exceeding the probability level of the most severe earthquake designated in major earthquakes or significant projects outlined in the zoning map. Consequently, these findings are recommended to be incorporated into the seismic reinforcement of urban planning in the Shen-Fu New District.

## Data Availability

The datasets generated during the current study are available from the corresponding author on request.
